# Adjunctive hyperbaric oxygen therapy in the management of severe lower limb soft tissue injuries: a systematic review

**DOI:** 10.1007/s00068-023-02426-2

**Published:** 2024-02-22

**Authors:** Esmee Kwee, Marieke Borgdorff, Tim Schepers, Jens A. Halm, Hay A. H. Winters, Robert P. Weenink, Milan L. Ridderikhof, Georgios F. Giannakópoulos

**Affiliations:** 1 Trauma Unit, Department of Surgery, Amsterdam UMC (Location AMC), J1A-214 Meibergdreef 9, 1105 AZ Amsterdam, The Netherlands; 2 Department of Plastic, Reconstructive Surgery and Handsurgery, Amsterdam UMC, Amsterdam, The Netherlands; 3 Department of Hyperbaric Medicine, Amsterdam UMC, Amsterdam, The Netherlands; 4 Department of Anesthesiology, Amsterdam UMC, Amsterdam, The Netherlands; 5 Department of Emergency Medicine, Amsterdam UMC, Amsterdam, The Netherlands

**Keywords:** Hyperbaric oxygenation, Traumatic injuries, Wound and injuries, Open fractures, Lower extremity

## Abstract

**Purpose:**

Traumatic crush injuries of the lower limb often accompany severe complications. The incorporation of hyperbaric oxygen therapy to standard trauma care may have the potential to diminish injury-related complications and improve outcome in such cases. This systematic review aims to evaluate the effectiveness of hyperbaric oxygen therapy in the management of severe lower limb soft tissue injuries.

**Methods:**

The electronic databases Medline, Embase and Cochrane Library were searched to identify studies involving patients with crush-associated sever lower limb soft tissue injuries who received hyperbaric oxygen therapy in conjunction with standard trauma care. Relevant data on type of injury, hyperbaric oxygen therapy protocol and outcome related to wound healing were extracted.

**Results:**

In total seven studies met the inclusion criteria, involving 229 patients. The studies included two randomized clinical trials, one retrospective cohort study, three case series and one case report. The randomized placebo-controlled clinical trial showed a significant increase in wound healing and decrease in the need for additional surgical interventions in the patient group receiving hyperbaric oxygen therapy when compared to those undergoing sham therapy. The randomized non-placebo-controlled clinical trial revealed that early hyperbaric oxygen therapy reduces tissue necrosis and the likelihood of long-term complications. The retrospective cohort study indicated that hyperbaric oxygen therapy effectively reduces infection rates and the need for additional surgical interventions. The case series and case report presented beneficial results with regard to wound healing when hyperbaric oxygen therapy was added to the treatment regimen.

**Conclusion:**

Hyperbaric oxygen therapy is generally considered a safe therapeutic intervention and seems to have a beneficial effect on wound healing in severe lower limb soft tissue injuries when implemented as an addition to standard trauma care.

**Supplementary Information:**

The online version contains supplementary material available at 10.1007/s00068-023-02426-2.

## Introduction

Traumatic crush injuries to the lower limb impose substantial burdens, comprising in psychosocial, physical and emotional implications [1]. These injuries encompass damage to various structures within the lower limb, including soft tissue, bone and neurovascular structures. The extent of lower limb soft tissue injury and contamination leads to an increased risk of complications, such as wound infection, osteomyelitis, wound necrosis and non-union [2–4]. Managing these complex injuries requires a comprehensive approach that addresses both immediate interventions and long-term functional and psychosocial outcomes.

Hyperbaric oxygen therapy (HBOT) has emerged as a potential adjunctive intervention in the management of limb injuries to diminish injury complications and improve outcome [5]. HBOT is administered in a monoplace or multiplace hyperbaric chamber, in which a patient breathes 100% oxygen supplied at a pressure greater than that at sea level. As a result of this hyperoxygenation, oxygen diffusion to tissues increases, contributing to the wound healing process [6–8]. Furthermore, HBOT reduces edema formation through vasoconstriction, diminishes inflammatory processes, increases collagen synthesis and angiogenesis, and inhibits the biochemical events in ischemia–reperfusion injury [9]. HBOT is regarded as a safe treatment modality with mostly avoidable or self-limiting side-effects. The most common side-effect of HBOT is middle ear barotrauma and the most feared adverse event is oxygen toxicity seizure [10].

Although prior research has predominantly focused on the role of HBOT in the treatment of chronic wounds, there is growing evidence supporting the beneficial results of HBOT in managing acute traumatic ischemias as well [7, 8, 11, 12]. Given the above-mentioned pathophysiological mechanisms HBOT has on wound healing, it is plausible that HBOT holds potential in addressing acute traumatic ischemia in the context of severe lower limb injuries. Therefore, the aim of this study is to present an overview of the available literature on the addition of HBOT in the management of crush-associated severe lower limb soft tissue injuries and to evaluate its effectiveness.

## Methods

### Literature search

The methods and results of this systematic review are written in accordance to the Preferred Reporting Items of Systematic Reviews and Meta-Analyses (PRISMA) statement [13–15]. The electronic bibliographic databases Medline, Embase and Cochrane Library were searched from inception up to November 21st 2022 by a clinical librarian. The search employed Mesh/Emtree search terms associated with ‘oxygen inhalation therapy’, ‘hyperbaric oxygen therapy’, ‘therapy, lower extremities, lower extremities’, ‘lower limbs’ and ‘wound healing’. The full electronic search strategy is detailed in Appendix A.

### Study selection

After duplicate removal, two reviewers (EK, GG) independently screened the titles and abstracts of the articles in the web-tool Rayyan [16]. The full texts of the studies were requested, and two reviewers (EK, GG) performed analysis of these articles to identify the final selection. In case the full text of an article could not be obtained, abstracts were allowed to be included. Whenever there was no consensus between the reviewers, a third reviewer (MB) assisted in the discussion.

During the selection, all studies on the addition of HBOT to standard trauma care in patients with crush-associated severe lower limb soft tissue injuries were included. Reviews, animal studies, studies in children, conference abstracts, poster presentations and non-English articles were excluded.

From the included articles more relevant studies were identified through reference list screening.

### Data extraction and quality scoring

During the data collection process, two reviewers (EK, GG) analyzed the final articles in detail. The following data was extracted: age, sex, type and severity of injury, time to surgery after injury, time to HBOT, HBOT protocols and follow-up time. All primary outcome measures on wound healing were extracted from the data, as well as secondary outcome measures: time until complete wound healing, incidence of wound complications and need for additional surgical interventions. The study characteristics, patient characteristics and results were entered into an electronic spreadsheet. Again, if consensus was not reached, a third reviewer (MB) assisted in the discussion.

Two reviewers (EK, GG) independently screened the included articles for risk of bias using the ROBINS-I tool for non-randomized studies and the Cochrane risk of bias tool for randomized controlled studies [17, 18]. Any discrepancies were resolved by consulting a third senior author (MB).

### Data synthesis

A descriptive overview was provided on the outcomes of the studies. The quantitative results were described separately for each study. If we required to calculate P values that were not reported in the original publications, this was done with a Z-score test for two proportions. P < 0.05 was regarded as statistically significant.

## Results

**Fig. 1:**
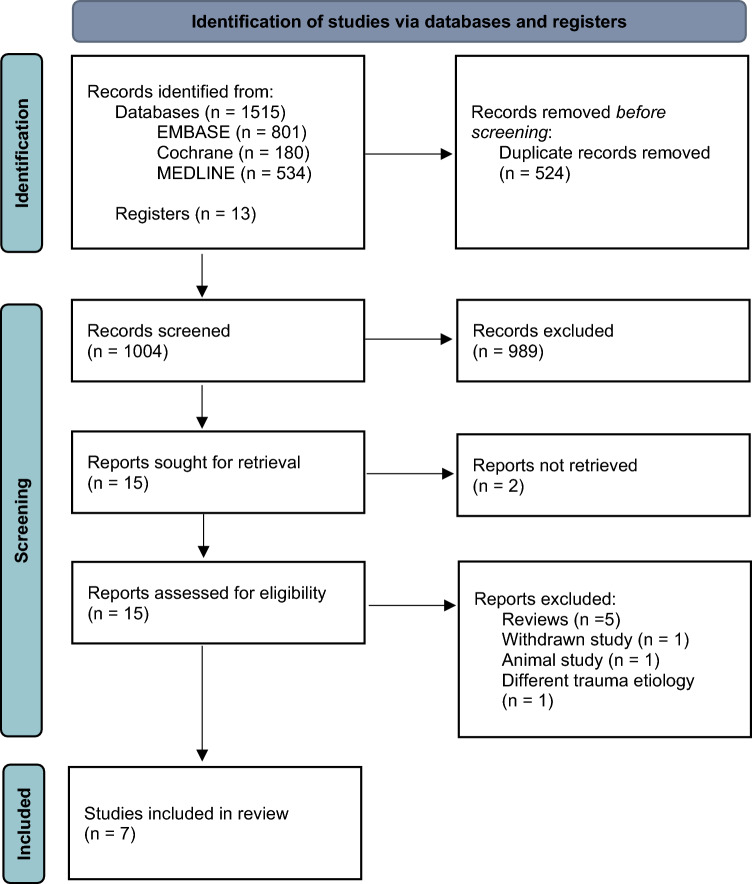
PRISMA flow chart of the included articles

The number of articles found was 1528, following duplicate removal 1004 articles remained. After analyzing the abstracts and full texts of the studies, a final selection of seven articles was made [19–25]. One article could not be obtained in full text but was included in the review based on the high relevance as judged from the abstract [22]. A flow chart of the inclusion process is shown in Fig. 1.

The study characteristics of the seven included studies are outlined in Table 1. The assessment of potential bias is detailed in Tables 2 and 3. The review included two randomized clinical trials [21, 25], one retrospective cohort study [23], three case series [19, 20, 22] and one case report [24]. The total study population size consisted of 229 patients, with 138 patients in the HBOT group and 91 patients in the control group. The mean age varied between 23.3 and 55 years. HBOT protocols exhibited variations across the studies, with pressure levels ranging between 2.3 and 2.8 ATA. Most studies used a HBOT protocol of two HBOT sessions a day with a duration of 90 min per session.

**Table 1:** Study Characteristics

Author (Year)	Study	HBOT size	Control size	Intervention & Control	HBOT Protocol	HBOT Sessions (mean)	Follow up (mths)
Monies-Chass et al. (1977) [19]	Case series	7	0	Standard care + HBOT	2 h with 4-h intervals, 2.8 ATA	9.5	NS
Shupak et al. (1987) [20]	Case series	13	0	Standard care + HBOT	Twice daily 90 min, 2.4 ATA	5.07	3–53
Bouachour et al. (1996) [21]	Randomized Placebo-controlled Clinical Trial	18	18	Intervention: standard care + HBOT Control: standard care + sham HBOT	Intervention: twice daily 90 min, 6dys, 2.5 ATA Control: idem, but at 1.1 ATA breathing air	12	NS
Matos et al. (1999) [22]	Case series	23	0	Standard care + HBOT	Twice daily 90 min, 2.36 ATA	12	24
Yamada et al. (2014) [23]	Retrospective cohort	16	13	Intervention: standard care + HBOT Control: standard care	Once daily 60 min, 2 ATA	6	12
Stefanidou et al. (2014) [24]	Case report	1	0	Standard care + HBOT	Twice daily 40 min, 2.4 ATA. First 3dys 4 sessions daily	32	7
Millar et al. (2022) [25]	Randomized Non-placebo-controlled Clinical Trial	60	60	Intervention: standard care + HBOT Control: standard care	12 sessions over 9 days. One session of 80 to 100 min, 2.4 or 2.8 ATA	12	24

*HBOT* Hyperbaric Oxygen Therapy, *ATA* atmosphere absolute, *yrs* years, *mths* months, *dys* days, *h* hours, *min* minutes, *NS* not specified

**Table 2:** ROBINS-I for non-randomised studies

Author	Confounding	Patient selection	Classification of interventions	Deviation from interventions	Missing data	Measurements errors	Selective reporting	Overall Risk of Bias
Monies-Chass et al. [19]	Moderate	Serious	Moderate	Moderate	Moderate	Serious	Moderate	Moderate
Shupak et al. [20]	Moderate	Moderate	Moderate	Low	Serious	Moderate	Moderate	Moderate
Matos et al. [22]	Serious	Moderate	Serious	Serious	Serious	Unclear	Unclear	Serious
Yamada et al. [23]	Moderate	Low	Moderate	Moderate	Moderate	Moderate	Moderate	Moderate
Stefanidou et al. [24]	Moderate	Low	Low	Low	Moderate	Low	Moderate	Moderate
Overall	Serious	Serious	Moderate	Moderate	Serious	Moderate	Moderate

**Table 3:** Quality assessment using Cochrane risk of bias tool

	Bouachour et al. [21]	Millar et al. [25]
Selection bias Random sequence generation	Unclear	Low
Selection bias Allocation concealment	Unclear	Unclear
Reporting bias Selective reporting	Unclear	Unclear
Other bias Other sources of bias	Low	Low
Performance bias Blinding (participants and personnel)	Low	High
Detection bias Blinding (outcome assessment)	Unclear	Low
Attrition bias Incomplete outcome data	Unclear	Low

Three studies were comparative studies, hence incorporating a control group [21, 23, 25]. Among these, Bouachour et al. and Miller et al. conducted randomized clinical trials. The study by Bouachour et al. was a placebo-controlled clinical trial, where sham hyperbaric treatment was applied in the control group, using a similar protocol as the intervention group but with a slightly increased pressure (1.1 ATA), and air instead of oxygen. The trial by Miller et al. followed a non-placebo-controlled design.

The results of the individual studies are presented in Table 4, including the following study outcomes: complete wound healing, incidence of necrosis, incidence of infection, additional surgical interventions, hospitalization and time to wound healing.

**Table 4:** Study Outcomes

	Outcome		
	HBOT	C	
**Complete Wound Healing**			
Monies-Chass et al. [19]	86%	X	
Shupak et al. [20]	62%	X	
Bouachour et al. [21]	94%	56%	P < 0.01
Matos et al. [22]	87%	X	
Stefanidou et al. [24]	100%	X	
**Incidence of Necrosis**			
Bouachour et al. [21]	6%	44%	P = 0.007
Millar et al. [25]	29%	53%	P = 0.01
Incidence of Infection			
Yamada et al. [23]	0%	46%	P = 0.003
Millar et al. [25]	22%	32%	P > 0.05
**Additional Surgical Techniques**			
Bouachour et al. [21]	6%	33%	P < 0.05
Yamada et al. [23]	0%	38%	P = 0.013
Millar et al. [25]	67%	56%	P > 0.05
**Hospitalization** (days)			
Bouachour et al. [21]	22.4	22.9	P > 0.05
Yamada et al. [23]	49	42.6	P > 0.05
Millar et al. [25]	15	15	P > 0.05
**Time to wound healing **(days)			
Bouachour et al. [21]	50.2	55.8	P > 0.05

*HBOT* Hyperbaric Oxygen Therapy, *C* control group, *X* no control group

### Complete wound healing

**Fig. 2:**
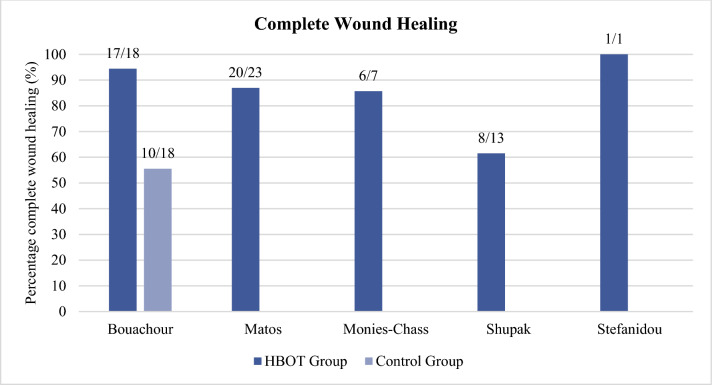
Complete Wound Healing

Wound healing was the primary outcome measure in most studies. The results are demonstrated in Fig. 2. Bouachour et al. defined complete wound healing as wound healing without tissue necrosis requiring surgical excision and demonstrated significantly higher rates of wound healing in the HBOT group when compared to the control group (94% vs. 56%, P < 0.01). Matos et al. achieved complete wound healing in 20 out of 23 patients (87%). Monies-Chass et al. reported complete wound healing in six out of seven patients (86%), with one patient requiring toe amputation due to residual ischemia distally despite the beneficial effects of HBOT. Shupak et al. reported complete wound healing in eight out of 13 patients (62%), a reduction of ischemia distally in four patients and no improvement in one patient. Stefanidou et al. reported complete wound healing in their case report.

### Incidence of necrosis

Bouachour et al. reported significantly lower rates of necrosis in the HBOT group when compared to the control group (5.6% vs. 44%, P = 0.007). Millar et al. demonstrated reduced necrosis in the HBOT group when compared to the control group (29% vs. 53%, P = 0.01) at 14-day assessment.

### Incidence of infection

Yamada et al. reported an infection rate of zero percent in the HBOT group versus 46% of the patients in the control group (P = 0.003). Millar et al. did not find statistically significant differences in acute infection rates between the HBOT group (22%) and the control group (32%) at 14-day assessment, and similar results were observed at 12-month assessment for the incidence of deep infections (8% vs. 15%, respectively).

### Additional surgical interventions

Bouachour et al. reported a significantly reduced need for additional surgical interventions in the HBOT group when compared to the sham HBOT group (5.6% vs. 33.3%, P < 0.05). Yamada et al. reported no need for additional surgical interventions in the HBOT group, while 38% of the patients in the control group required additional surgical interventions (P = 0.013). Millar et al. found that 67% in the HBOT group required additional surgical interventions, compared to 56% in the control group, with the difference being non-significant.

### Hospitalization

Length of hospital stay was assessed in three studies, no significant differences in outcomes between the HBOT group and the control group were observed.

### Time to wound healing

Bouachour et al. did not find any significant differences between the HBOT group and the control group regarding the time to wound healing.

## Discussion

This review summarizes the literature on the effectiveness of HBOT on outcome in traumatic lower limb soft tissue injuries. Based on our results, we can conclude that HBOT when added to standard trauma care holds potential to positively influence wound healing.

Past studies have mainly researched the effects of HBOT in chronic wounds rather than acute injuries, even though evidence on the role of HBOT in the management of acute wounds does exist. The systematic review conducted by Garcia et al. studied the addition of HBOT in the management of crush injuries and traumatic ischemias, including studies published between 1966 and 2003 [26]. The review encompassed diverse injuries, such as upper and lower limb injuries as well as finger traumas and compartment syndromes, with varying mechanisms of injury. In an attempt to address the issue of heterogeneity, we decided to restrict our search to the inclusion of crush-associated lower limb soft tissue injuries. By doing so, we intended to steer towards more specific recommendations on its management.

The positive effects of HBOT on the pathophysiological processes in acute traumatic ischemias, especially on the ischemia, edema and hypoxia triad, are well described in current literature. Despite these findings, HBOT has not yet found its place in guidelines for the management of traumatic injuries, including lower limb injuries. One of the reasons for this, is the lack in large randomized clinical trials considering this research topic. Only two of the studies included in this review are randomized clinical trials. It is therefore tempting to call for more trials, and indeed the highest grade of evidence should always be sought. However, several issues preclude conduction of large trials on this subject. Firstly, severe lower limb injury is a heterogeneous clinical entity, and design of a clinical trial that includes a well-defined patient population while at the same time ensuring an adequate inclusion rate is challenging. Secondly, the application of multiple sessions of HBOT may entail quite a large placebo effect. Inclusion of a control group that undergoes sham HBOT may help to limit bias in this regard. Lansdorp et al. described the design of sham HBOT protocols and the study by Bouachour et al. showed that it is possible [27]. However, for many hyperbaric centers’ treatment with sham HBOT means that their only hyperbaric chamber is blocked for significant amounts of time, reducing availability of ‘real’ HBOT and increasing costs. Also, as Millar et al. pointed out, sham HBOT could be seen as a negative intervention, because it removes patients from regular clinical care for considerable amounts of time [25]. Moreover, the scarcity of hyperbaric chambers in general adds to the difficulty of conducting large-scale trials with HBOT. Moon et al. reported that less than ten percent of all hyperbaric treatment facilities in the United States provide continuous availability for emergency HBOT [28].

We believe that in the field of hyperbaric medicine it is important to make decisions based on all available evidence, and not only rely on large trials, which – for the reasons stated above – are difficult to perform. Evidence from pathophysiology, case reports, observational studies, and randomized clinical trials should be synthetized. Even though our study includes only seven studies in total, of which two were clinical trials, we still felt an important need to perform this review and to draw conclusions from it.

Future studies should prioritize the development of standardized protocols for HBOT in the management of severe lower limb injuries, enhancing the reproducibility of the treatment and facilitating comparisons between the studies. Combing these standardized protocols with data from snapshot audits could advance the understanding of HBOT’s efficacy in severe lower limb injuries [29].

### Limitations

This review holds several limitations. Firstly, the included studies lack comprehensive data, notably regarding follow-up duration, thereby complicating the evaluation of long-term functional and psychosocial outcomes after HBOT and after standard care alone. Also, the unavailability of the full text of Matos et al. resulted in the inclusion of an abstract, compromising the quality of this study due to the inability to access all information. Moreover, the majority of included studies were small case reports and series including a relatively limited number of patients, potentially influencing observed effects and restricting the generalizability of findings. Furthermore, variations in time from injury until surgery, the initiation of the first HBOT session, as well as differences in HBOT protocols and number of HBOT sessions among the studies, contributed to existing heterogeneity. At last, it is important to note that, except for Millar et al., the remaining studies span a decade or more, during which standard trauma care significantly evolved. This evolution potentially reduces the relevance of older findings, posing a challenge in weighing their outcomes. Despite these limitations, this systematic review provides novel insights into the treatment of acute lower limb injuries with HBOT.

## Conclusion

Based on the seven studies included in this review, the addition of HBOT to standard trauma care seems to have a beneficial effect on wound healing in crush-associated severe lower limb soft tissue injuries.

### Supplementary Information

Below is the link to the electronic supplementary material.

Supplementary file1 (DOCX 24 KB)

## References

[1] de Andrade Fonseca M, Cordeiro Matias AG, de Freitas Gomes ML, Almeida Matos M (2019). Impact of lower limb fractures on the quality of life. Ortop Traumatol Rehabil.

[2] Meling T, Harboe K, Søreide K (2009). Incidence of traumatic long-bone fractures requiring in-hospital management: a prospective age- and gender-specific analysis of 4890 fractures. Injury.

[3] Court-Brown CM, Bugler KE, Clement ND, Duckworth AD, McQueen MM (2012). The epidemiology of open fractures in adults: a 15-year review. Injury.

[4] Harris AM, Althausen PL, Kellam J, Bosse MJ, Castillo R, Lower Extremity Assessment Project (LEAP) Study Group (2009). Complications following limb-threatening lower extremity trauma. J Orthop Trauma.

[5] Mathieu D, Marroni A, Kot J (2017). Tenth European consensus conference on hyperbaric medicine: recommendations for accepted and non-accepted clinical indications and practice of hyperbaric oxygen treatment. Diving Hyperb Med.

[6] Niinikoski JH (2004). Clinical hyperbaric oxygen therapy, wound perfusion, and transcutaneous oximetry. World J Surg.

[7] Buettner MF, Wolkenhauer D (2007). Hyperbaric oxygen therapy in the treatment of open fractures and crush injuries. Emerg Med Clin North Am.

[8] Eskes A, Ubbink DT, Lubbers M, Lucas C, Vermeulen H (2010). Hyperbaric oxygen therapy for treating acute surgical and traumatic wounds. Cochrane Database Syst Rev.

[9] De Wolde SD, Hulskes RH, Weenink RP, Hollmann MW, Van Hulst RA (2021). The effects of hyperbaric oxygenation on oxidative stress, inflammation and angiogenesis. Biomolecules.

[10] Heyboer M, Sharma D, Santiago W, McCulloch N (2017). Hyperbaric oxygen therapy: side effects defined and quantified. Adv Wound Care (New Rochelle).

[11] Sharma R, Sharma SK, Mudgal SK, Jelly P, Thakur K (2021). Efficacy of hyperbaric oxygen therapy for diabetic foot ulcer, a systematic review and meta-analysis of controlled clinical trials. Sci Rep.

[12] Dauwe PB, Pulikkottil BJ, Lavery L, Stuzin JM, Rohrich RJ (2014). Does hyperbaric oxygen therapy work in facilitating acute wound healing: a systematic review. Plast Reconstr Surg.

[13] Moher D, Liberati A, Tetzlaff J, Altman DG, PRISMA Group (2009). Preferred reporting items for systematic reviews and meta-analyses: the PRISMA statement. PLoS Med.

[14] Higgins JPT, Altman DG (eds) (2008) Cochrane handbook for systematic reviews of interventions, Version 5.0.1 (updated September 2008). The Cochrane Collaboration. https://www.cochrane-handbook.org/.

[15] https://www.crd.york.ac.uk/PROSPERO/#recordDetails

[16] Ouzzani M, Hammady H, Fedorowicz Z, Elmagarmid A (2016). Rayyan-a web and mobile app for systematic reviews. Syst Rev.

[17] Sterne JAC, Hernán MA, Reeves BC, Savović J, Berkman ND, Viswanathan M, Henry D, Altman DG, Ansari MT, Boutron I, Carpenter JR, Chan AW, Churchill R, Deeks JJ, Hróbjartsson A, Kirkham J, Jüni P, Loke YK, Pigott TD, Ramsay CR, Regidor D, Rothstein HR, Sandhu L, Santaguida PL, Schünemann HJ, Shea B, Shrier I, Tugwell P, Turner L, Valentine JC, Waddington H, Waters E, Wells GA, Whiting PF, Higgins JPT (2016). ROBINS-I: a tool for assessing risk of bias in non-randomized studies of interventions. BMJ.

[18] Deeks JJ, Higgins JPT, Altman DG, on behalf of the Cochrane Statistical Methods Group and the Cochrane Bias Methods Group (eds) Chapter 9: Analysing data and undertaking meta-analyses. In: Higgins JPT, Green S (eds). Cochrane Handbook for Systematic Reviews of Interventions Version 5.1.0 [updated March 2011]. The Cochrane Collaboration, 2011. Available from www.cochrane-handbook.org.

[19] Monies-Chass I, Hashmonai M, Hoere D, Kaufman T, Steiner E, Schramek A (1977). Hyperbaric oxygen treatment as an adjuvant to reconstructive vascular surgery in trauma. Injury.

[20] Shupak A, Gozal D, Ariel A (1987). Hyperbaric oxygenation in acute peripheral posttraumatic ischemia. J Hyperbaric Med.

[21] Bouachour G, Cronier P, Gouello JP, Toulemonde JL, Talha A, Alquier P (1996). Hyperbaric oxygen therapy in the management of crush injuries: a randomized double-blind placebo-controlled clinical trial. J Trauma.

[22] Matos LA, Hutson JJ, Bonet H (1999). HBO as an adjunct treatment for limb salvage in crush injuries of the extremities. Undersea Hyperb Med.

[23] Yamada N, Toyoda I, Doi T, Kumada K, Kato H, Yoshida S, Shirai K, Kanda N, Ogura S (2014). Hyperbaric oxygenation therapy for crush injuries reduces the risk of complications: research report. Undersea Hyperb Med.

[24] Stefanidou S, Kotsiou M, Mesimeris T (2014). Severe lower limb crush injury and the role of hyperbaric oxygen treatment: a case report. Diving Hyperb Med.

[25] Millar IL, Lind FG, Jansson KÅ, Hájek M, Smart DR, Fernandes TD, McGinnes RA, Williamson OD, Miller RK, Martin CA, Gabbe BJ, Myles PS, Cameron PA, HOLLT investigator group (2022). Hyperbaric oxygen for lower limb trauma (HOLLT): an international multi-centre randomised clinical trial. Diving Hyperb Med.

[26] Garcia-Covarrubias L, McSwain NE, Van Meter K, Bell RM (2005). Adjuvant hyperbaric oxygen therapy in the management of crush injury and traumatic ischemia: an evidence-based approach. Am Surg.

[27] Lansdorp CA, van Hulst RA (2018). Double-blind trials in hyperbaric medicine: a narrative review on past experiences and considerations in designing sham hyperbaric treatment. Clin Trials.

[28] Butler FK, Moon RE (2020). Emergency hyperbaric oxygen therapy: a service in need of resuscitation - an open letter. Undersea Hyperb Med.

[29] Bass GA, Kaplan LJ, Ryan ÉJ, Cao Y, Lane-Fall M, Duffy CC, Vail EA, Mohseni S (2023). The snapshot audit methodology: design, implementation and analysis of prospective observational cohort studies in surgery. Eur J Trauma Emerg Surg.

