# Supplementary material for: Adjunctive hyperbaric oxygen therapy in the management of severe lower limb soft tissue injuries: a systematic review
Source: Eur J Trauma Emerg Surg. 2024 Feb 22;50(3):1093–100. doi: 10.1007/s00068-023-02426-2 (PMC11249575; doi:10.1007/s00068-023-02426-2)
Supplement: Supplementary file 1 — Supplementary file1 (DOCX 24 KB) [file 68_2023_2426_MOESM1_ESM.docx]

**Appendix A. Full electronic search strategy**

MEDLINE (via OVID):

Database(s): Ovid MEDLINE(R) ALL 1946 to October 20, 2020
Search Strategy:

| # | Searches | Results |
| --- | --- | --- |
| 1 | oxygen inhalation therapy/ or hyperbaric oxygenation/ or Oxygen/ae, tu, th or Atmospheric Pressure/ or Atmosphere Exposure Chambers/ | 39160 |
| 2 | (hyperbar* or HBO or HBOT or high pressure oxygen* or 100% oxygen*).ti,ab,kf. | 18084 |
| 3 | ((monoplace or multiplace) adj5 chamber*).ti,ab,kf. | 182 |
| 4 | 1 or 2 or 3 | 47239 |
| 5 | Extremities/ or exp lower extremity/ or ankle fractures/ or tibial fractures/ or exp Crush Injuries/ | 207251 |
| 6 | (limb* or extremit* or foot or feet or ankle* or heel* or buttock* or hip* or knee* or leg* or tibia* or ALI or acute limb isch*).ti,ab,kf. | 1235038 |
| 7 | (crush* adj6 (injur* or trauma* or fractur*)).ti,ab,kf. | 4689 |
| 8 | 5 or 6 or 7 | 1299434 |
| 9 | exp Wound Healing/ | 127360 |
| 10 | cicatrix.ti,ab,kf. | 1423 |
| 11 | (wound* adj6 (heal* or clos*)).ti,ab,kf. | 89256 |
| 12 | 9 or 10 or 11 | 179297 |
| 13 | 4 and 8 and 12 | 440 |

EMBASE (via OVID):

Database(s): Embase Classic+Embase 1947 to 2020 October 20
Search Strategy:

| # | Searches | Results |
| --- | --- | --- |
| 1 | oxygen therapy/ or hyperbaric oxygen therapy/ or oxygen/th or atmospheric pressure/ or atmosphere exposure chamber/ | 52648 |
| 2 | (hyperbar* or HBO or HBOT or high pressure oxygen* or 100% oxygen*).ti,ab,kw. | 25269 |
| 3 | ((monoplace or multiplace) adj5 chamber*).ti,ab,kw. | 213 |
| 4 | 1 or 2 or 3 | 74088 |
| 5 | limb/ or exp lower limb/ or exp leg bone/ or limb injury/ or exp leg injury/ or exp limb fracture/ or crush trauma/ | 764217 |
| 6 | (limb* or extremit* or foot or feet or ankle* or heel* or buttock* or hip* or knee* or leg* or tibia* or ALI or acute limb isch*).ti,ab,kw. | 1690525 |
| 7 | (crush* adj6 (injur* or trauma* or fractur*)).ti,ab,kw. | 6263 |
| 8 | 5 or 6 or 7 | 1956678 |
| 9 | exp wound healing/ or wound closure/ | 183899 |
| 10 | cicatrix.ti,ab,kw. | 1358 |
| 11 | (wound* adj6 (heal* or clos*)).ti,ab,kw. | 124783 |
| 12 | 9 or 10 or 11 | 228968 |
| 13 | 4 and 8 and 12 | 623 |

[Cochrane Database of Systematic Reviews](https://www.cochranelibrary.com/), [Cochrane Central Register of Controlled Trials](https://www.cochranelibrary.com/)

Issue 10 of 12, October 2020

ID Search Hits

#1 MeSH descriptor: [Oxygen Inhalation Therapy] explode all trees 1539

#2 MeSH descriptor: [Atmospheric Pressure] explode all trees 360

#3 MeSH descriptor: [Atmosphere Exposure Chambers] explode all trees 81

#4 (hyperbar* or HBO or HBOT or high pressure oxygen* or 100% oxygen*):ti,ab,kw 11492

#5 (monoplace or multiplace) near/5 chamber* 48

#6 #1 or #2 or #3 or #4 or #5 12715

#7 (limb* or extremit* or foot or feet or ankle* or heel* or buttock* or hip* or knee* or leg* or tibia* or ALI or acute limb isch*):ti,ab,kw 123555

#8 MeSH descriptor: [Lower Extremity] explode all trees 7041

#9 MeSH descriptor: [Extremities] this term only 346

#10 MeSH descriptor: [Crush Injuries] explode all trees 17

#12 (crush* near/9 (injur* or trauma* or fractur*)):ti,ab,kw 141

#13 #7 or #8 or #9 or #10 or #12 123896

#14 MeSH descriptor: [Wound Healing] explode all trees 5823

#15 (wound* near/6 (heal* or clos*)):ti,ab,kw 13272

#16 (cicatrix):ti,ab,kw 1009

#17 #14 or #15 or #16 14481

#18 #6 and #13 and

**Update 21-10-2020 to 20-05-2022**

MEDLINE (via OVID):

Database(s): Ovid MEDLINE(R) and Epub Ahead of Print, In-Process, In-Data-Review & Other Non-Indexed Citations and Daily 1946 to May 19, 2022
Search Strategy:

| # | Searches | Results |
| --- | --- | --- |
| 1 | oxygen inhalation therapy/ or hyperbaric oxygenation/ or Oxygen/ae, tu, th or Atmospheric Pressure/ or Atmosphere Exposure Chambers/ | 41286 |
| 2 | (hyperbar* or HBO or HBOT or high pressure oxygen* or 100% oxygen*).ti,ab,kf. | 19103 |
| 3 | ((monoplace or multiplace) adj5 chamber*).ti,ab,kf. | 195 |
| 4 | 1 or 2 or 3 | 49816 |
| 5 | Extremities/ or exp lower extremity/ or Tibial Fractures/ or exp Crush Injuries/ | 220668 |
| 6 | (limb* or extremit* or foot or feet or ankle* or heel* or buttock* or hip* or knee* or leg* or tibia* or ALI or acute limb isch*).ti,ab,kf. | 1358307 |
| 7 | (crush* adj6 (injur* or trauma* or fractur*)).ti,ab,kf. | 5040 |
| 8 | 5 or 6 or 7 | 1424634 |
| 9 | exp Wound Healing/ | 138205 |
| 10 | cicatrix.ti,ab,kf. | 1465 |
| 11 | (wound* adj6 (heal* or clos*)).ti,ab,kf. | 103738 |
| 12 | 9 or 10 or 11 | 199137 |
| 13 | 4 and 8 and 12 | 492 |
| 14 | limit 13 to yr="2020 -Current" | 74 |

EMBASE (via OVID):

Database(s): **Embase Classic+Embase**1947 to 2022 May 19
Search Strategy:

| # | Searches | Results |
| --- | --- | --- |
| 1 | oxygen therapy/ or hyperbaric oxygen therapy/ or oxygen/th or atmospheric pressure/ or atmosphere exposure chamber/ | 61979 |
| 2 | (hyperbar* or HBO or HBOT or high pressure oxygen* or 100% oxygen*).ti,ab,kw. | 26655 |
| 3 | ((monoplace or multiplace) adj5 chamber*).ti,ab,kw. | 223 |
| 4 | 1 or 2 or 3 | 83915 |
| 5 | limb/ or exp lower limb/ or exp leg bone/ or limb injury/ or exp leg injury/ or exp limb fracture/ or crush trauma/ | 835845 |
| 6 | (limb* or extremit* or foot or feet or ankle* or heel* or buttock* or hip* or knee* or leg* or tibia* or ALI or acute limb isch*).ti,ab,kw. | 1842015 |
| 7 | (crush* adj6 (injur* or trauma* or fractur*)).ti,ab,kw. | 6595 |
| 8 | 5 or 6 or 7 | 2132184 |
| 9 | exp wound healing/ or wound closure/ | 202739 |
| 10 | cicatrix.ti,ab,kw. | 1314 |
| 11 | (wound* adj6 (heal* or clos*)).ti,ab,kw. | 137795 |
| 12 | 9 or 10 or 11 | 253540 |
| 13 | 4 and 8 and 12 | 742 |
| 14 | limit 13 to yr="2020 -Current" | 143 |

Cochrane Library: Cochrane Database of Systematic Reviews Issue 5 of 12, May 2022 2 hits

Cochrane Central Register of Controlled Trials Issue 4 of 12, April 2022 24 hits

ID Search Hits

#1 MeSH descriptor: [Oxygen Inhalation Therapy] explode all trees 1730

#2 MeSH descriptor: [Atmospheric Pressure] explode all trees 383

#3 MeSH descriptor: [Atmosphere Exposure Chambers] explode all trees 81

#4 (hyperbar* or HBO or HBOT or high pressure oxygen* or 100% oxygen*):ti,ab,kw 13389

#5 (monoplace or multiplace) near/5 chamber* 54

#6 #1 or #2 or #3 or #4 or #5 14736

#7 (limb* or extremit* or foot or feet or ankle* or heel* or buttock* or hip* or knee* or leg* or tibia* or ALI or acute limb isch*):ti,ab,kw 142574

#8 MeSH descriptor: [Lower Extremity] explode all trees 7722

#9 MeSH descriptor: [Extremities] this term only 366

#10 MeSH descriptor: [Crush Injuries] explode all trees 31

#12 (crush* near/9 (injur* or trauma* or fractur*)):ti,ab,kw 169

#13 #7 or #8 or #9 or #10 or #12 142946

#14 MeSH descriptor: [Wound Healing] explode all trees 6293

#15 (wound* near/6 (heal* or clos*)):ti,ab,kw 15145

#16 (cicatrix):ti,ab,kw 1175

#17 #14 or #15 or #16 16505

#18 #6 and #13 and #17 with Cochrane Library publication date Between Oct 2020 and May 2022 26

**UPDATE: 20-5-2022 t/m 21-11-2022**

**21-11-2022:**

| Databases: |  |  |
| --- | --- | --- |
| Medline (Ovid), Embase (Ovid), Cochrane Library | Before duplicate removal | After duplicate removal |
| Totaal | 71 | 41 |

MEDLINE (via OVID):

Database(s): Ovid MEDLINE(R) and Epub Ahead of Print, In-Process, In-Data-Review & Other Non-Indexed Citations and Daily 1946 to November 18, 2022
Search Strategy:

| # | Searches | Results |
| --- | --- | --- |
| 1 | oxygen inhalation therapy/ or hyperbaric oxygenation/ or Oxygen/ae, tu, th or Atmospheric Pressure/ or Atmosphere Exposure Chambers/ | 41695 |
| 2 | (hyperbar* or HBO or HBOT or high pressure oxygen* or 100% oxygen*).ti,ab,kf. | 19420 |
| 3 | ((monoplace or multiplace) adj5 chamber*).ti,ab,kf. | 198 |
| 4 | 1 or 2 or 3 | 50434 |
| 5 | Extremities/ or exp lower extremity/ or Tibial Fractures/ or exp Crush Injuries/ | 223986 |
| 6 | (limb* or extremit* or foot or feet or ankle* or heel* or buttock* or hip* or knee* or leg* or tibia* or ALI or acute limb isch*).ti,ab,kf. | 1397251 |
| 7 | (crush* adj6 (injur* or trauma* or fractur*)).ti,ab,kf. | 5152 |
| 8 | 5 or 6 or 7 | 1463971 |
| 9 | exp Wound Healing/ | 140527 |
| 10 | cicatrix.ti,ab,kf. | 1475 |
| 11 | (wound* adj6 (heal* or clos*)).ti,ab,kf. | 108633 |
| 12 | 9 or 10 or 11 | 205047 |
| 13 | 4 and 8 and 12 | 501 |
| 14 | limit 13 to yr="2022 -Current" | 20 |

EMBASE (via OVID):

Database(s): **Embase Classic+Embase**1947 to 2022 November 18
Search Strategy:

| # | Searches | Results |
| --- | --- | --- |
| 1 | oxygen therapy/ or hyperbaric oxygen therapy/ or oxygen/th or atmospheric pressure/ or atmosphere exposure chamber/ | 65023 |
| 2 | (hyperbar* or HBO or HBOT or high pressure oxygen* or 100% oxygen*).ti,ab,kw. | 27122 |
| 3 | ((monoplace or multiplace) adj5 chamber*).ti,ab,kw. | 228 |
| 4 | 1 or 2 or 3 | 87186 |
| 5 | limb/ or exp lower limb/ or exp leg bone/ or limb injury/ or exp leg injury/ or exp limb fracture/ or crush trauma/ | 859433 |
| 6 | (limb* or extremit* or foot or feet or ankle* or heel* or buttock* or hip* or knee* or leg* or tibia* or ALI or acute limb isch*).ti,ab,kw. | 1894452 |
| 7 | (crush* adj6 (injur* or trauma* or fractur*)).ti,ab,kw. | 6720 |
| 8 | 5 or 6 or 7 | 2192613 |
| 9 | exp wound healing/ or wound closure/ | 209216 |
| 10 | cicatrix.ti,ab,kw. | 1316 |
| 11 | (wound* adj6 (heal* or clos*)).ti,ab,kw. | 143388 |
| 12 | 9 or 10 or 11 | 262269 |
| 13 | 4 and 8 and 12 | 771 |
| 14 | limit 13 to yr="2022 -Current" | 35 |

Cochrane Library:

[Cochrane Database of Systematic Reviews](https://www.cochranelibrary.com/) Issue 11 of 12, November 2022 1 hit

[Cochrane Central Register of Controlled Trials](https://www.cochranelibrary.com/) Issue 10 of 12, October 2022 15 hits

ID Search Hits

#1 MeSH descriptor: [Oxygen Inhalation Therapy] explode all trees 1755

#2 MeSH descriptor: [Atmospheric Pressure] explode all trees 385

#3 MeSH descriptor: [Atmosphere Exposure Chambers] explode all trees 81

#4 (hyperbar* or HBO or HBOT or high pressure oxygen* or 100% oxygen*):ti,ab,kw 14245

#5 (monoplace or multiplace) near/5 chamber* 56

#6 #1 or #2 or #3 or #4 or #5 15602

#7 (limb* or extremit* or foot or feet or ankle* or heel* or buttock* or hip* or knee* or leg* or tibia* or ALI or acute limb isch*):ti,ab,kw 152153

#8 MeSH descriptor: [Lower Extremity] explode all trees 7845

#9 MeSH descriptor: [Extremities] this term only 368

#10 MeSH descriptor: [Crush Injuries] explode all trees 38

#12 (crush* near/9 (injur* or trauma* or fractur*)):ti,ab,kw 187

#13 #7 or #8 or #9 or #10 or #12 152540

#14 MeSH descriptor: [Wound Healing] explode all trees 6392

#15 (wound* near/6 (heal* or clos*)):ti,ab,kw 15894

#16 (cicatrix):ti,ab,kw 1237

#17 #14 or #15 or #16 17300

#18 #6 and #13 and #17 with Cochrane Library publication date Between May 2022 and Nov 2022 16
